# New Method of Administering Chloroform

**Published:** 1896-02

**Authors:** 


### New Method of Administering Chloroform.

Preferring, in general, chloroform to ether M. Rosenberg (*Berlin klin Woch.*, No. 10, 1895), finds that the accidents due to chloroform are to be attributed to the manner of administering and not to the drug itself. As every one knows, the danger of chloroform anæsthetic is the arrest of the heart, or of the respiration. Both, he contends, are brought about reflexly by the irritating action of the chloroform upon the terminations of trigeminus distributed to the mucous membrane of the nose. The same reflex can be produced by any other anæsthetic taken through the nose. To obviate this he first renders the mucous membrane of the nose anæsthetic by the use of cocaine, which in itself is an antidote to chloroform. As a result of his experience of this method in fifty cases he concludes as follows:

1. The commencement of anæsthesia is less disagreeable for the patient, who never makes defensive movements.

2. The excitement stage is often wanting, and is always slight except in the case of alcoholics.

3. During anæsthesia it is very rarely the patient vomits, and if vomiting does occur there is little retching.

4. Upon awakening, the patient experiences no disagreeable sensation, and is not haunted by the smell of chloroform or ether.

The following is his routine practice: A few minutes before the general anæsthesia the patient is directed to blow the nose strongly so as to clear the mucous membrane of mucus, and leaning forward or sitting (never lying down), is directed to snuff in each nostril a centigramme of powder consisting of some inert substance and 10 percent of cocaine hydrochlorate. This is repeated in about three minutes, and general anæsthesia is commenced. If the operation be prolonged the insufflation is repeated at intervals of half an hour. It is also repeated when the operation is over, as it causes the patient to waken up more rapidly. As to the mode of administering the chloroform itself, the author is strongly in favor of the continuous administration, drop by drop.—*Canadian Practitioner.*

